# The Effect of Traditional Chinese Mind-Body Exercise (Baduanjin) and Brisk Walking on the Dorsal Attention Network in Older Adults With Mild Cognitive Impairment

**DOI:** 10.3389/fpsyg.2019.02075

**Published:** 2019-09-10

**Authors:** Rui Xia, Pingting Qiu, Huiying Lin, Bingzhao Ye, Mingyue Wan, Moyi Li, Jing Tao, Lidian Chen, Guohua Zheng

**Affiliations:** ^1^College of Rehabilitation Medicine, Fujian University of Traditional Chinese Medicine, Fuzhou, China; ^2^Department of Rehabilitation, The First Affiliated Hospital of Nanchang University, Nanchang, China; ^3^Fujian Key Laboratory of Rehabilitation Technology, Fuzhou, China; ^4^College of Nursing and Health Management, Shanghai University of Medicine and Health Sciences, Shanghai, China

**Keywords:** mind-body exercise, Baduanjin, brisk walking, mild cognitive impairment, dorsal attention network

## Abstract

A growing number of studies have shown that mind-body exercise is beneficial to cognitive function, especially memory, in elderly MCI patients. However, few studies have explored the effect of mind-body exercise on the attention of MCI population. We recruited 69 participants and divided them equally into Baduanjin, brisk walking (BWK) exercise or usual physical activity (UAP) control groups. The two exercise groups performed 60 min of exercise three times per week for 24 weeks. All subjects underwent whole-brain functional MRI and assessment of attentional abilities, including selective, divided, and sustained attention, and processing speed at baseline and after 24 weeks. The results show that: Baduanjin exercise significantly increased the selective attention of MCI patients, and Dorsal attention network (DAN) of Baduanjin exercise group exhibited functional connectivity decreased in right rolandic operculum (ROL. R), right middle temporal gyrus (MTG. R), right supramarginal inferior parietal, angular gyri (IPL. R), right precuneus (PCUN. R), and right fusiform gyrus (FFG. R) regions compared with the other two groups. The BWK exercise group had obviously functional connectivity increased in IPL. R and decreased in the MTG. R region compared to that in the UAP group. But no significant association between the changes of functional connectivity of DAN and the change of attentional ability test was observed. Thus, our data indicated Baduanjin exercise may be a potential beneficial intervention to improve the attention of the elderly with MCI. Further study with more samples is necessary to elucidate its imaging mechanism.

## Introduction

At present, over 46 million people are living with dementia, and by 2050, this number will rise to almost 131.5 million worldwide; dementia has been identified as a global health priority due to the growing burden of the disease (Prince, 2015). Dementia is a progressive disease with a long duration. Pathological changes start taking place up to 20 years before the presentation of clinical symptoms. The currently available medications for dementia only alleviate the disease’s symptoms but are not able to stop or slow its progression. Therefore, there is an urgent need for strategies to prevent people from developing dementia (Prince et al., 2016). Mild cognitive impairment (MCI) is a transitional state between normal aging and dementia, accompanied by the decline of memory, attention and other cognitive domains. People with MCI constitute a group at high risk for dementia, and MCI is increasingly prevalent among older age groups (Petersen, 2016). There is accumulating evidence for a breakdown in processes related to attention in older adults with MCI and in the early stages of dementia (Duchek and Balota, 2005; Belleville et al., 2008). Studies have shown that attention deficits in MCI patients may be the entry point for developing dementia and can be an early indication of subsequent changes in other cognitive functions (Marra et al., 2000; Zhou and Wang, 2005). Thus, early identification and intervention related to attention impairment in patients with MCI is very important.

Some recent studies have shown attention to be a key cognitive domain that benefits from physical activity (Iuliano et al., 2015; Condello et al., 2017). Brain function, as measured by functional magnetic resonance imaging (fMRI), has shown that physical activity is associated with increased activity in left dorsolateral prefrontal, posterior parietal, and anterior cingulate cortices (Rosano et al., 2010), which are important components of the core region of the dorsal attention network (DAN) (Kim, 2014). The DAN is consistently involved in attention and has been associated with working memory and episodic memory encoding (Majerus et al., 2018; Stawarczyk et al., 2018). Recent studies have found intranetwork and internetwork functional disruptions in the DAN in MCI patients (Qian et al., 2015; Bi et al., 2018). Therefore, some studies have suggested that functional change in the DAN could be used as a sensitive indicator of MCI disease progression (Li et al., 2012; Zhang et al., 2015). Given the relationship between attention and the DAN, it can be speculated that the DAN is a promising avenue by which increasing physical activity can promote the attention abilities of patients with MCI. However, previous measurements of physical activity have been imprecise, often in the form of self-reports, and have failed to provide an accurate picture for some specific types of physical activity.

Baduanjin is a traditional Chinese mind-body aerobic exercise of moderate intensity and is one of the most common forms of Qigong that has been practiced in China for over 1000 years. Unlike other types of aerobic exercise, practitioners are required to achieve coordination between mind and body when they are practicing Baduanjin exercise (Kim et al., 2016). Studies have demonstrated that regular practice of Baduanjin exercise can not only result in physiological benefits, such as improved cardiopulmonary function, balance, and reduced osteoarthritis, but also actually improve cognitive function in older people with or without cognitive impairment (Zou et al., 2018). However, no studies have reported the effect of Baduanjin exercise on attentional ability or the relationship between attention and the DAN in MCI patients.

## Materials and Methods

### Participants

Participants in this study came from a randomized controlled trial, for which a total of 135 MCI patients were recruited from Fuzhou, China. Based on a 1:1:1 ratio, participants were randomly assigned to a Baduanjin group, a brisk walking (BWK) group and a usual activity control group with 45 participants each group (Zheng et al., 2016a). A total of 69 participants underwent baseline attention tests and MRI scans, of which 60 completed the post-intervention attention tests and MRI scans (20 individuals in each group). Inclusion criteria for study participants were an age of 60 years or older; conformity with Peterson’s MCI diagnostic criteria (Petersen, 2004); and no regular physical exercise for at least half a year (regular exercise means exercise with a frequency of at least twice a week and at least 20 min per session).

This trial was approved by the Medical Ethics Committee of the Second People’s Hospital of Fujian Province (approval number 2014-KL045-02). All participants provided written informed consent prior to participation.

### Intervention Protocol

Baduanjin exercise Baduanjin (BDJ) exercise intervention was conducted according to the standards promulgated by the General Administration of Sport in China in 2003, which consisted of 10 postures (including the beginning and ending posture) (Health Qigong Management Center of General Administration of Sport of China, 2003). Participants gathered at a community center at 7 am and practiced Baduanjin supervised by a coach. Baduanjin training was assigned at two community centers (Cangxia and Longfeng Community Center in Fuzhou City) with 20–25 individuals per center. The exercise intervention lasted 24 weeks with a frequency of three sessions a week and 60 min per session. In addition, participants, including those in the usual physical activity (UAP) group, also received a health education program, which included instruction regarding cognitive disorders, healthy eating and living habits. Two professional coaches from Fujian University of Traditional Chinese Medicine (FJTCM) were employed to guide participants’ training.

Brisk walking Participants received 24 weeks of BWK training at a frequency of three sessions a week and 60 min per session in addition to the health education courses described above. The intensity of exercise was controlled such that heart rate remained in the range of 55–75% of the maximum reserve heart rate (Ying, 2012).

Usual physical activity control Participants in the UAP group were instructed to maintain their original physical activity habits and only received health education training at a frequency of one session every 8 weeks, 30 min per session.

### Attentional Ability Measurement

According to the Diagnostic and Statistical Manual of Mental Disorders, Fifth Edition (DSM-V) recommendations, attentional ability encompassing the four dimensions of selective attention, divided attention, sustained attention and processing speed were measured at baseline and after intervention in this study by using the Color-Word Matching Stroop task (cwmStroop), the divided and sustained attention Test of Attention Performance software (TAP V.2.3, Vera Fimm, Psychologische Testsysteme), and the digit-symbol coding task (DSC), respectively (Gong, 1983; American Psychiatric Association, 2013). cwmStroop task created in E-Prime software (version 2.0, Psychology Software Tools, Inc., Sharpsburg, PA, United States), two lines of text appear on the screen, participants are asked to determine whether the coloring of the first line of text matches the meaning of the next line as soon as possible, and to record the correct number and reaction time under three conditions. During the divided attention subtask, participants must consider both visual (mobile cross) and auditory (low and high) stimuli. When there are four intersections forming a square or when the sound interrupts alternately, press the response button as soon as possible. Record the correct number of reactions and the number of reaction times. During the sustained attention subtask, participants should keep an eye on the various graphics on the screen and press the button quickly when two consecutive identical graphics appear. DSC requires participants to convert numbers into corresponding symbols as soon as possible in 90 s.

### fMRI Scan Parameters and Data Preprocessing

Magnetic resonance imaging (MRI) scans were obtained at baseline and after intervention using a 3.0-Tesla General Electric scanner (Milwaukee, WI, United States) with an eight-channel phased-array head coil. T1-weighted images were collected using a three-dimensional magnetization-prepared rapid acquisition gradient-echo (3DMPRAGE) sequence with the following parameters: echo time = min, field of view = 240 × 240 mm, flip angle = 15°, inversion time = 450 ms, slice thickness = 1 mm, and 164 slices per acquisition. An echo planar imaging sequence was performed for resting-state scans with the following parameters: repetition time = 2100 ms, echo time = 30 ms, echo spacing = 20 ms, field of view = 200 × 200 mm, flip angle = 90°, slice thickness = 3 mm with a 0.6-mm gap, voxel size = 3.125 × 3.125 × 3.6 mm^3^, 42 slices, 64 × 64 matrix, phases per location = 160, bandwidth = 31.2, slice order: [1,3,…41,2,4,…42], Subjects were required to stay awake with their eyes closed and ears plugged during the MRI scan.

The preprocessing of the fMRI data was performed using the toolbox for Data Processing and Analysis for Brain Imaging, Version 2.3 (DPABI) in MATLAB (MathWorks, Inc., Natick, MA, United States). The preprocessing steps included slice-timing, realignment, coregistration, segmentation, spatial normalization, smoothing, and filtering. In this study, 42 slices of interlace scanning were implemented, and the second slice in the middle of scanning sequence was used as the reference slices for slice-timing. The Friston-24 model is used for head motion correction, and the data of participants with three-dimensional translation >3 mm and/or three-dimensional rotation >3 degrees are eliminated. Through linear transformation, the structure image is transformed into the function image space to match and overlay each other. Linear and quadratic trends are used as regression variables to remove low frequency drift of BOLD signals. Then use DARTEL tool to convert functional data from an individual native space to MNI space. Finally, performed spatial smoothing (FWMH kernel: 4 mm) and temporal filtering (0.01–0.1 Hz) to improve the signal-to-noise ratio (Yan et al., 2016).

### Statistical Analysis

#### Behavioral Data Analysis

Behavioral data at baseline and after intervention were analyzed using the SPSS 24.0 (IBM, Chicago, IL, United States) software package. One-way analysis of variance (ANOVA) or a non-parametric test was applied to compare differences across the three groups. *Post hoc* tests (Bonferroni correction) were applied to explore between-group differences. Statistical significance was defined as two-sided *P* < 0.05. The scores of cognitive data pre-post change was further calculated, and covariance analysis was conducted. At the same time, gender, age, and education level were adjusted as covariates.

#### Resting-State fMRI Data Analysis

Independent component analysis (ICA) and the Group ICA/IVA of fMRI Toolbox (GIFT^1^, version 4.0 a) were applied to analyze the MRI data. Performed the Infomax algorithm to group spatial ICA, and a Gaussian ICA method was used to back reconstruct components (Calhoun et al., 2001). The *Z-*value is a correlation coefficient between the time series of each single voxel and the time series of an independent component. Higher *Z*-value indicates stronger functional connectivity. Converting the correlation coefficient between time series of each voxels and independent components into *Z*-value, and the strength of functional connection is judged accordingly. The DAN was identified according to the rules reported in a previous study (Choe et al., 2015), and group statistics were performed in which we extracted the Z map from two components then reduced the *Z*-values before and after the intervention (pre-post-intervention). Results of the rs-fMRI data were corrected for multiple comparisons in group level and voxel level, one-way ANOVA was performed to analyze the changes in the *Z*-values across the three groups, and a *post hoc* test with the least-significance-difference (LSD) method was applied to analyze the between-group differences. Voxel level corrected using the AlphaSim correction, thresholds of voxel-wise *P* < 0.01, cluster-level *P* < 0.05 (Ke et al., 2018).

In addition, to explore the association between the changes of functional connectivity in the DAN and changes of behavioral factors, we performed a multiple regression analysis including age, gender, and education as covariates.

## Results

### Baseline Characteristics

Sixty participants completed MRI scans at baseline and after the intervention with 20 participants each group. There were no significant differences in the demographic data or the Montreal Cognitive Assessment (MoCA), Global Deterioration Scale level and Geriatric Depression Scale scores at baseline across the three groups (Table 1).

**TABLE 1:** Baseline characteristics of the participants (mean ± SD).

**Characteristics**	**BDJ (*n* = 23)**	**BWK (*n* = 23)**	**UPA (*n* = 23)**	***F*(χ^2^)**	***P***
Age (year)	65.79 ± 4.35	64.88 ± 3.30	65.86 ± 5.28	0.488	0.783
Gender (male/female)	6/17	11/12	6/17	3.261	0.196
Average years of education (year)	11.22 ± 3.45	10.48 ± 2.47	11.35 ± 3.54	0.696	0.706
Handedness (right/left)	23/0	23/0	23/0	–	–
MoCA	22.30 ± 2.40	21.65 ± 2.35	20.83 ± 3.27	1.724	0.186
GDS^1^ grade II/III, n	16/7	13/10	11/12	2.260	0.323
GDS^2^ scores	6.09 ± 2.98	4.57 ± 2.68	5.26 ± 2.75	1.694	0.192

*BDJ, Baduanjin exercise group; BWK, brisk walking group; UPA, usual physical activity control group. ^1^Global deterioration scale. ^2^Geriatric depression scale.*

### The Effect of Baduanjin Exercise on Attentional Ability

There was no significant difference in performance on the selective attention, divided attention, sustained attention or processing speed measures at baseline across the three groups. After the 24 weeks of the intervention period, covariance analysis pre-post changes of attention with adjusted gender, age and education showed only average number of correct congruent condition were significantly different between the three groups (*P* = 0.038); further *post hoc* analysis showed that the average number of correct congruent condition for the Baduanjin group was significant increase than that of the UAP group (*P* = 0.008). The group differences on other parameters of selective attention, divided attention, sustained attention and processing speed were not found (Table 2).

**TABLE 2:** Comparison of attention ability among groups.

**Outcomes**	**Groups**	**Numbers of participants**	**Baseline**		**Pre-post changes^∗^**	
			**Mean @ SD**	***P-*value**	**Mean @ SE**	***P-*value**
**Selective attention (stroop test)**						
Neutral reaction time (ms)	BDJ	23	1029.45 ± 113.04	0.681	−15.79 ± 20.91	0.918
	BWK	23	1039.48 ± 80		−16.96 ± 11.41	
	UPA	23	1052.55 ± 69.42		−3.45 ± 23.56	
Neutral correct number (times)	BDJ	23	44.48 ± 12.78	0.484	5.30 ± 2.20	0.340
	BWK	23	45.48 ± 7.95		3.61 ± 1.51	
	UPA	23	41.48 ± 13.46		0.20 ± 2.12	
Congruent reaction time (ms)	BDJ	23	1007.77 ± 111.61	0.941	−11.35 ± 20.34	0.225
	BWK	23	999.38 ± 75.48		0.48 ± 18.84	
	UPA	23	1006.28 ± 68.38		71.73 ± 29.06	
Congruent correct number (times)	BDJ	23	45.22 ± 13.48	0.690	6.55 ± 2.71	0.038^1^
	BWK	23	47.96 ± 8.79		2.12 ± 2.09	
	UPA	23	45.65 ± 11.92		−6.6 ± 3.55	
Incongruent reaction time (ms)	BDJ	23	1068.65 ± 121.71	0.326	31.34 ± 20.69	0.219
	BWK	23	1097.09 ± 93.22		−36.93 ± 25.63	
	UPA	23	1118.99 ± 122.54		−44.83 ± 51.18	
Incongruent correct number (times)	BDJ	23	29.48 ± 13.41	0.564	7.60 ± 2.55	0.264
	BWK	23	31.13 ± 11.1		6.53 ± 3.24	
	UPA	23	27.3 ± 11.72		−4.40 ± 3.83	
**Divided attention (TAP test)**						
Auditory reaction time (ms)	BDJ	23	720.65 ± 156.57	0.070	−8.15 ± 44.23	0.248
	BWK	23	718.30 ± 417.39		−105.71 ± 114	
	UPA	23	810.35 ± 282.23		−53.60 ± 69.05	
Auditory correct number (times)	BDJ	23	14.17 ± 3.08	0.213	−0.25 ± 0.93	0.633
	BWK	23	14.09 ± 3.60		1.29 ± 0.99	
	UPA	23	12.87 ± 3.43		−0.60 ± 1.73	
Visual reaction time (ms)	BDJ	23	1028.57 ± 202.12	0.116	−43.95 ± 80.26	0.586
	BWK	23	978.95 ± 230.55		−51.65 ± 54.61	
	UPA	23	1050.82 ± 181.61		8.20 ± 45.69	
Visual correct number (times)	BDJ	23	13.83 ± 3.75	0.754	0.50 ± 1.32	0.319
	BWK	23	14.32 ± 3.47		0.94 ± 0.52	
	UPA	23	14.55 ± 3.43		−0.40 ± 0.45	
**Sustained attention (TAP test)**						
Reaction time (ms)	BDJ	23	685.35 ± 132.21	0.094	4.50 ± 43.66	0.641
	BWK	23	624.00 ± 98.79		−32.55 ± 56.86	
	UPA	23	702.09 ± 119.08		−58.87 ± 42.45	
Correct number (times)	BDJ	23	39.52 ± 8.84	0.550	−3.50 ± 1.88	0.145
	BWK	23	39.91 ± 9.94		1.00 ± 2.65	
	UPA	23	38.17 ± 8.18		3.07 ± 2.49	
**Processing speed (DSC test)**						
DSC scores	BDJ	23	33.22 ± 10.63	0.675	4.50 ± 1.22	0.867
	BWK	23	34.65 ± 10.14		2.94 ± 1.28	
	UPA	23	31.89 ± 7.27		4.13 ± 2.02	

*BDJ, Baduanjin exercise group; BWK, brisk walking group; UPA, usual physical activity contrl group. DSC, digit–symbol coding test. *Post hoc* test: ^1^*P_BDJ vs.BWK_* = 0.442, *P_BDJ vs. UPA_* = 0.008; *P_BWK vs. UPA_* = 0.117. ^∗^Covariance analysis pre-post changes of attention with adjusted gender, age, and education level.*

#### Dorsal Attention Network Changes

**FIGURE 1:**
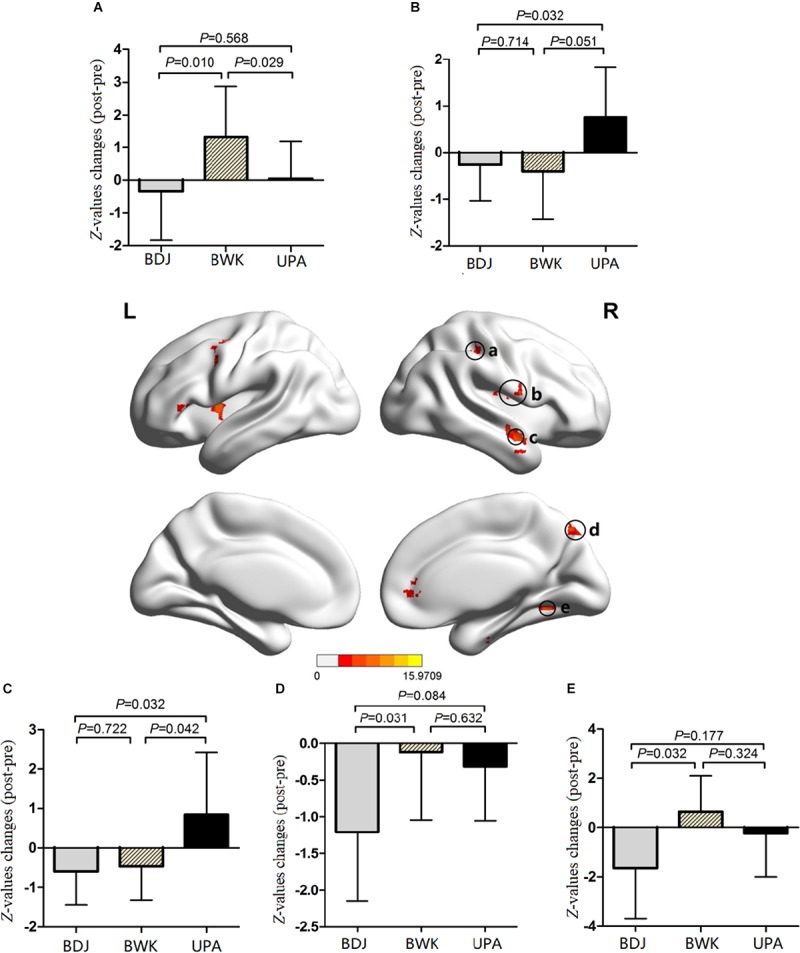
One-way ANOVA and *post hoc* analysis results of DAN. One-way ANOVA showed that, after the 24-week exercise, there were significant difference at five regions in DAN (cluster-corrected at Alphasim *P* < 0.05; voxels *P* < 0.01): IPL.R(a), ROL.R(b), MTG.R(c), PCUN.R(d), and FFG.R(e). *Post hoc* analysis results are shown, compared to the brisk walking (BWK) group or usual physical activity (UAP) group, in these five regions in Baduanjin group had obvious negative activation, and all the *Z*-values changes were lower than other two groups. **(A)** The *Z*-values changes of BWK group appeared a positive change, significantly higher than the other two groups. **(B)** There were significant negative changes in Baduanjin group than UAP group. **(C)** The *Z*-values changes of UAP group appeared a positive change, significantly higher than the other two groups. **(D)** The negative changes were occurred across three groups, and there was significant difference between Baduanjin group and BWK group. **(E)** The *Z*-values changes of BWK group appeared a positive change, significantly higher than the Baduanjin group.

The results of the ICA are presented in Table 3. One-way ANOVA showed that after the 24-week exercise period, there were significant differences in the DAN in five regions (cluster-corrected at AlphaSim *P* < 0.05; voxels *P* < 0.01): right middle temporal gyrus (MTG. R), right fusiform gyrus (FFG. R), right rolandic operculum (ROL. R), right precuneus (PCUN. R), and right supramarginal inferior parietal and angular gyri (IPL.R). The results of *post hoc* analysis applied Bonferroni correction have no statistical difference among the three groups, and the results applied LSD method were shown in Figure 1. Compared to the BWK group or UAP group, IPL. R, ROL. R, MTG. R, PCUN. R, and FFG. R exhibited obvious functional connectivity reduced in the Baduanjin group, and all changes in *Z*-value were smaller than those in the other two groups (all *P* < 0.05). In IPL. R, the change in *Z*-value for the BWK group were functional connectivity increased and were significantly greater than that in the other two groups (*P_BWK vs. BDJ_* = 0.001, *P_BWK vs. UPA_* = 0.029). In ROL. R, the change in *Z*-value were significantly more functional connectivity reduced in the Baduanjin group than in the UAP group (*P* = 0.032). In MTG. R, the change in *Z*-value in the UAP group were functional connectivity increased and were significantly greater than that in the other two groups (*P_UPA vs. BDJ_* = 0.032, *P_UPA vs. BWK_* = 0.042). In PCUN. R, the functional connectivity reduced were significantly different between the Baduanjin group and the BWK group (*P* = 0.031). In FFG. R, the change in *Z*-value for the BWK group were functional connectivity increased and were significantly greater than that for the Baduanjin group (*P* = 0.032) (Figure 1).

**TABLE 3:** Regions show significant functional connectivity changes with the DAN across three groups.

**Regions**	**Abbr.**	**MNI coordinates**			**Peak *z*-value**	**Cluster size (voxels)**
		***x***	***y***	***z***		
Middle temporal gyrus R	MTG.R	57	0	−15	7.521	101
Fusiform gyrus R	FFG.R	30	−57	−9	8.5847	61
Superior frontal gyrus, medial R	ORB supmed.R	0	54	−6	7.8328	52
Insula L	INS.L	−36	0	3	9.062	58
Rolandic operculum R	ROL.R	45	−18	15	9.5218	62
Precentral gyrus L	PreCG.L	−36	−3	51	8.9678	49
Precuneus R	PCUN.R	15	−78	48	8.3251	48
Inferior parietal, but supramarginal and angular gyri R	IPL.R	39	−48	42	8.8844	58

*Alphasim correction, *P* < 0.05, Minimum voxels collection = 46.*

To investigate the association between those functional connectivity changes and the correct congruent condition changes, we performed a multiple regression analysis adjusted with age, gender and education. We found no significant association between those functional connectivity changes and the average number of congruent condition changes across all participants (*P* > 0.05) (Table 4).

**TABLE 4:** Regression analyses between functional connectivity changes and the correct congruent condition changes.

**ROI_*S*_**	**Correct congruent condition changes**		
	**Beta**	**Standardized beta**	***P*-values**
MTG.R	4.603	0.273	0.872
FFG.R	18.754	0.269	0.516
ROL.R	6.69	0.008	0.819
PCUN.R	6.73	0.058	0.817
IPL.R	14.126	0.199	0.628

*MTG.R, middle temporal gyrus R; FFG.R, fusiform gyrus R; ROL.R, rolandic operculum R; PCUN.R, precuneus R; IPL.R, inferior parietal, but supramarginal and angular gyri R. Adjusted gender, age, and education level.*

## Discussion

In this study, we investigated differences in attentional ability at baseline and after 24 weeks of Baduanjin practice compared with a BWK group and a UAP group in older adults with MCI. We found that compared to the UAP group, the Baduanjin group improved with average number of correct congruent condition on a test of selective attention ability. ICA showed that Baduanjin exercise group exhibited functional connectivity decreased in ROL. R, MTG. R, IPL. R, PCUN. R, and FFG. R region of DAN compared with the other two groups. The BWK exercise group had obviously functional connectivity increased in IPL. R and decreased in the MTG. R region compared to that in the UAP group. However, regression analysis does not show the significant association between attentional ability change and DAN functional connectivity change. Our data suggest that Baduanjin exercise has a potential benefit on improving the selective attention of MCI patients, but it still is uncertain that this potential effect may be related to changes in the functional connectivity of the DAN.

Accumulating evidence suggests that aerobic exercise has a positive effect on cognitive function in MCI patients (Zheng et al., 2016b; Sáez de Asteasu et al., 2017). Cognitive function can be divided into memory, attention, executive function and other domains. Currently, most studies focus on memory, but few focuses on whether exercise is beneficial to the attentional ability of patients with MCI. The results of a randomized controlled study showed that a moderate-intensity walking program, which consisted of two sessions a week for **1** year, decreased the reaction time in a Stroop task for women with MCI (Van Uffelen et al., 2008). Another study also showed that moderate aerobic exercise for 16 weeks significantly improved reaction time on Stroop incongruent and interference conditions in older individuals (Coetsee and Terblanche, 2017). Witte’s research showed that there was a significant improvement in divided attention after a 5-month karate training (Witte et al., 2016). Consistent with previous studies, the results of this study also showed that accuracy on Stroop congruent tasks for the Baduanjin group was significantly improved over that of the **UAP** group. Although previous studies have shown that aerobic exercise enhances sustained attention by stimulating noradrenergic activity and that this effect may be related to exercise intensity and exercise type (Radel et al., 2018), this study did not find that **BWK** or Baduanjin exercise improved sustained attention in MCI patients.

Baduanjin, a traditional Chinese mind-body aerobic exercise with a lower intensity of exercise, yields the improvement in the selective attention domain in MCI patients. This result is similar to a previous study, which found that traditional Chinese mind-body exercise can improve Stroop task performance in older adults (Ji et al., 2017). According to the theory of traditional Chinese medicine, Baduanjin exercise involves a practice of mind-body integration to cultivate “*qi*” to maximize both physical and mental wellbeing. Furthermore, Baduanjin only includes eight movements and is more suitable than Tai Chi for cognitive deficits in older individuals (Xiong et al., 2015). Previous studies reported that Baduanjin had an improved effect on global cognitive function and memory in older adults (Tao et al., 2016, 2017a,b). Therefore, the findings of this study that Baduanjin may be beneficial to selective attention ability in MCI patients are consistent.

In Alzheimer’s disease patients, the DAN and the ventral attention network have been shown to be completely impaired, while MCI patients suffered from severe DAN damage, but ventral attention network function was relatively preserved (Zhang et al., 2015). The DAN is a task-positive network, and it has an anticorrelated functional relationship with the default mode network (DMN). In the resting state, the DMN has been shown to be activated, but the DAN was inactivated. As age increases, this anticorrelation was reduced and more pronounced in MCI patients (Spreng et al., 2016; Esposito et al., 2018). Significant differences have been observed in the functional connectivity of the DAN between MCI patients and normal older control subjects, in regions such as the left FEF, left IPS, dorsomedial frontal lobe and posterior cingulate gyrus (Bi et al., 2018). One study demonstrated that abnormal brain regions in the DAN were associated with cognitive impairment in MCI patients (Tao et al., 2017a). Electrical or magnetic stimulation of the DAN core brain areas has been shown to improve attention in patients with MCI (Anderkova et al., 2018; Siddiqi et al., 2019). Aerobic exercise increases the frontal gray matter, including the anterior cingulate cortex, the supplemental motor area, middle frontal gyrus, superior frontal gyrus, and the superior and inferior parietal lobules, and its functional connections, which may be involved in top-down attentional processes (Colcombe et al., 2003, 2004). Our research showed that the functional connectivity of ROL. R, MTG. R and PCUN. R exhibited negative activation in the BWK group and that these changes may be beneficial in maintaining the anticorrelated functional relationship between the DAN and DMN. Additionally, this study showed that the functional connectivity of IPL. R, ROL. R, MTG. R, PCUN. R, and FFG. R exhibited more significant negative activation after 24 weeks of Baduanjin training. Baduanjin is a mind-body exercise, different from conventional aerobic exercise, and it also encompasses components of mindfulness and meditation, which require more attention and control. Mindfulness and meditation can improve attention in older individuals and ameliorate the coupling among the DAN, self-referential processes and affective responses (Froeliger et al., 2012; Doll et al., 2016). However, our study does not find the significant association between the negative activation of DAN and the change of attentional ability which may be related to the small sample size of this study or the short duration of intervention. Therefore, further study with more samples is necessary to elucidate its imaging mechanism.

There are several potential limitations to this study. First, the sample size of this study was so small that were not able to perform stringent multiple comparison corrections. Second, there were no thresholds for attentional impairment in the inclusion criteria, which led to heterogeneity in the participants’ attentional abilities. Third, attention is a complex topic, and current measurement metrics may be limited. For example, the Stroop test has been used to evaluate selective attention in some studies and to reflect executive control function in other studies, which introduces confusion regarding the use of the Stroop test as an outcome measurement method. In addition, the intensity of Baduanjin exercise cannot be quantified, and the quality of the intervention may not be consistent, although we adopted strict quality control measures.

In summary, this study suggests that regular Baduanjin exercise might be potential beneficial to improve selective attentional abilities in patients with MCI, but current data does not thoroughly support that this effect is related to changes in the functional connectivity of the DAN. Further researches with more samples are necessary to clarify the effects of Baduanjin on the attentional abilities of patients with MCI, and the imaging mechanism in the functional connectivity of brain network.

## Data Availability

The datasets used and/or analyzed during the current study are available from the corresponding author upon request.

## Author Contributions

LC and GZ conceived and designed the study. RX and GZ wrote the manuscript. JT was in charge of coordination and direct implementation. RX, PQ, HL, BY, MW, and ML managed the training location and the follow-up. All authors contributed to drafting the manuscript, and have read and approved the final manuscript.

## Conflict of Interest Statement

The authors declare that the research was conducted in the absence of any commercial or financial relationships that could be construed as a potential conflict of interest.

## Footnotes

1 http://icatb.sourceforge.net/
